# Closing the gap in our understanding of infectious diseases

**DOI:** 10.1186/s12879-023-08389-4

**Published:** 2023-06-16

**Authors:** Chester Kalinda, Elvis Temfack

**Affiliations:** 1 University of Global Health Equity (UGHE), Bill and Joyce Cummings Institute of Global Health (IGH), Kigali Heights, Plot 772 KG 7 Ave, PO Box 6955, Kigali, Rwanda; 2 Africa Centers for Disease Control and Prevention, Addis Ababa, Ethiopia

## Abstract

Systematic reviews (SR) and meta-analyses (MA) have become important in addressing specific questions of clinical importance and presenting evidence from an in-depth analysis of literature and aiding clinical decision-making. The “Systematic Reviews on infectious diseases” collection will address several important questions by summarizing large bodies of evidence in a reproducible and concise approach to advance our knowledge and understanding of infectious diseases.

## Introduction

Infectious diseases continue to be the primary source of morbidity and mortality in all patients globally [1]. An increase in the number of clinically indistinguishable pathogens [2], the rapid proliferation of multidrug-resistant pathogens and their severity underscore the difficulties associated with their management [2, 3]. This thus increases research needs to determine and understand the extent of interactions between the social and environmental determinants of health and how these interactions influence the risks of infectious disease outbreaks. The threats of emergence and re-emergence of infectious disease remain, and this may be partly explained by changes in the suitable ecological niches that alter species range and density thus increasing the risk of infectious diseases occurrence [4–6]. This suggests the need for proactive approaches in identifying the risk factors that favour infectious disease emergence and spread to enhance better preventive measures. This BMC Infectious Diseases collection ‘Systematic Reviews on infectious diseases’ will present research articles in the area of infectious diseases that will help enhance our understanding of infectious diseases while presenting strengths and weaknesses from the available evidence thus improving clinical decision-making.

Proactive approaches will need to be value-focused, while others will need to synthesize and analyze data from multiple studies to help provide evidence-based recommendations that address infectious disease problems and enhance healthcare outcomes. Furthermore, these approaches can also help identify important strategies that can be used by stakeholders to guide decision-making. Thus, systematic reviews and meta-analyses are important approaches that can help enhance our understanding and management of infectious diseases and aid in making informed decisions that can lead to improved health outcomes. Systematic reviews and meta-analyses integrate various and diverse outcomes while addressing the replicability of clinical evidence important to patients’ well-being. Several systematic reviews focusing on a single topic such as COVID-19 have been published in the last two years. These reviews have highlighted several knowledge gaps, proposed interventions for control and prevention and highlighted the need for further research to enhance clinical decision-making [7].

Systematic reviews and meta-analyses have become popular and gained prominence in research as they provide comprehensive and rigorous approaches to understanding some of the potential existing knowledge gaps. Furthermore, revisions to reporting guidelines such as the Preferred Reporting Items for Systematic Reviews and Meta-Analyses (PRISMA) statement to facilitate transparent and complete reporting of systematic reviews have been done [8, 9]. Frameworks for prospective, adaptive meta-analysis have also been developed to reduce the risks of potential bias and increase the reliability of systematic reviews to be done in a timely and thorough manner [10, 11]. In addition, the use of artificial intelligence and machine learning in systematic reviews and meta-analysis have been suggested to enhance efficiency and accuracy [12, 13]. All these developments indicate some of the advances in the methodological approaches to systematic and meta-analyses.

On the other hand, an increase in the number of systematic reviews and meta-analyses has also led to questions relating to the usefulness of their results [12]. This is because there have been several systematic reviews that have been done focusing on a single topic which leads to duplication of effort and waste of resources [14]. This suggests the importance of following community-established guidelines when conducting systematic reviews to reduce the potential redundancy of multiple systematic reviews on the same topic and also the risks of generating faulty interpretations when not done correctly. Systematic reviews and meta-analyses continue to make important contributions in bridging the gap in knowledge about certain phenomena and raising important methodological questions. However, there is a need to continue working on addressing the need for quality work and the usefulness of these studies without duplicating prior efforts.

This *BMC Infectious Diseases* collection ‘Systematic Reviews on infectious diseases’ will feature research articles that will draw our attention and attempt to unravel some of the complexities in understanding infectious diseases. This collection aims to have an in-depth understanding of the prevention, diagnosis, and management of infectious and sexually transmitted diseases in humans, as well as related molecular genetics, pathophysiology, and epidemiology. Emerging evidence suggests an increase in the outbreak of infectious diseases and STIs and their associated mortalities. These outbreaks pose a serious challenge to public health practitioners and researchers especially in low and middle-income settings where the burden of poverty and diseases remains high. To address these challenges, a better understanding of pathogen biology and disease transmission mechanisms, optimization of diagnostic tools and epidemiology of these diseases is important. Advances in all these which can help in significantly improving the diagnosis, prevention, and control of infectious diseases can be addressed by aggregating current knowledge in systematic reviews and meta-analyses to understand the insights that new evidence provides. In doing so, we would be closing the gap in our understanding of infectious diseases.

## Data Availability

Not applicable.

## References

[1] Lozano R, Naghavi M, Foreman K, Lim S, Shibuya K, Aboyans V, Abraham J, Adair T, Aggarwal R, Ahn SY (2012). Global and regional mortality from 235 causes of death for 20 age groups in 1990 and 2010: a systematic analysis for the global burden of Disease Study 2010. The lancet.

[2] Simner PJ, Miller S, Carroll KC (2017). Understanding the promises and Hurdles of Metagenomic Next-Generation sequencing as a Diagnostic Tool for Infectious Diseases. Clin Infect Dis.

[3] Bloom DE, Cadarette D (2019). Infectious disease threats in the twenty-first century: strengthening the global response. Front Immunol.

[4] Cunze S, Glock G, Kochmann J, Klimpel S (2022). Ticks on the move—climate change-induced range shifts of three tick species in Europe: current and future habitat suitability for Ixodes ricinus in comparison with Dermacentor reticulatus and Dermacentor marginatus. Parasitol Res.

[5] Koch LK, Cunze S, Kochmann J, Klimpel S (2020). Bats as putative Zaire ebolavirus reservoir hosts and their habitat suitability in Africa. Sci Rep.

[6] Mahmud AS, Martinez PP, He J, Baker RE (2020). The impact of climate change on vaccine-preventable diseases: insights from current research and new directions. Curr Environ health Rep.

[7] Silal SP (2021). Operational research: a multidisciplinary approach for the management of infectious disease in a global context. Eur J Oper Res.

[8] Page MJ, Moher D, Bossuyt PM, Boutron I, Hoffmann TC, Mulrow CD, Shamseer L, Tetzlaff JM, Akl EA, Brennan SE (2021). PRISMA 2020 explanation and elaboration: updated guidance and exemplars for reporting systematic reviews. BMJ.

[9] Page MJ, McKenzie JE, Bossuyt PM, Boutron I, Hoffmann TC, Mulrow CD, Shamseer L, Tetzlaff JM, Akl EA, Brennan SE (2021). The PRISMA 2020 statement: an updated guideline for reporting systematic reviews. BMJ.

[10] Ranganathan P, Aggarwal R (2021). Study designs: part 9 - Meta-analysis (II). Perspect Clin Res.

[11] Tierney JF, Fisher DJ, Vale CL, Burdett S, Rydzewska LH, Rogozińska E, Godolphin PJ, White IR, Parmar MKB (2021). A framework for prospective, adaptive meta-analysis (FAME) of aggregate data from randomised trials. PLoS Med.

[12] Raina SK, Kumar R (2022). Is typifying systematic reviews and meta-analysis as the top on the ladder justified?. J Family Med Prim Care.

[13] Kuo RY, Harrison C, Curran T-A, Jones B, Freethy A, Cussons D, Stewart M, Collins GS, Furniss D (2022). Artificial intelligence in fracture detection: a systematic review and meta-analysis. Radiology.

[14] Borges do Nascimento IJ, O’Mathúna DP, von Groote TC, Abdulazeem HM, Weerasekara I, Marusic A, Puljak L, Civile VT, Zakarija-Grkovic I, Pericic TP (2021). Coronavirus disease (COVID-19) pandemic: an overview of systematic reviews. BMC Infect Dis.

